# Expression, purification, crystallization and crystallographic study of *Lutzomyia longipalpis* LJL143

**DOI:** 10.1107/S2053230X15009486

**Published:** 2015-06-27

**Authors:** Alan Kelleher, Zhuyun Liu, Christopher A. Seid, Bin Zhan, Oluwatoyin A. Asojo

**Affiliations:** a National School of Tropical Medicine, Baylor College of Medicine, 1102 Bates Avenue, Suite 550, Mail Stop BCM320, Houston, TX 77030-3411, USA

**Keywords:** leishmaniasis, *Lutzomyia longipalpis*, neglected tropical diseases, diagnosis, sandfly, salivary proteins, transmission

## Abstract

Leishmaniasis is a neglected vector-borne disease with a global prevalence of over 12 million cases and 59 000 annual deaths. Transmission of the parasite requires salivary proteins, including LJL143 from the New World sandfly *Lutzomyia longipalpis*. LJL143 is a known marker of sandfly exposure in zoonotic hosts. LJL143 was crystallized from soluble protein expressed using *Pichia pastoris*. X-ray data were collected to 2.6 Å resolution from ortho­rhombic crystals belonging to space group *P*2_1_2_1_2_1_, with average unit-cell parameters *a* = 57.39, *b* = 70.24, *c* = 79.58 Å. The crystals are predicted to have a monomer in the asymmetric unit, with an estimated solvent content of 48.5%. LJL143 has negligible homology to any reported structures, so the phases could not be determined by molecular replacement. All attempts at S-SAD failed and future studies include experimental phase determination using heavy-atom derivatives.

## Abstract 2

LJL143, a salivary protein from *L. longipalpis*, was produced using *P. pastoris* and crystallized in space group *P*2_1_2_1_2_1_.

## 1. Introduction   

Leishmaniasis is a neglected tropical disease that is endemic in over 88 countries and is caused by over 20 species and subspecies of parasitic protozoa of the genus *Leishmania*. Clinical manifestations of leishmaniasis vary from self-healing skin lesions to fever, anemia, skin destruction and death (Pavli & Maltezou, 2010 ▸). There are four main clinical forms of leishmaniasis: visceral leishmaniasis, cutaneous leishmaniasis, mucocutaneous leishmaniasis and diffuse cutaneous leishmaniasis. Visceral leishmaniasis is the most serious form and can be fatal if left untreated. While most cases of cutaneous leishmaniasis are mild and heal without treatment, other forms of leishmaniasis are treated with pentavalent antimony drugs, including meglumine antimonate and sodium stibo­gluconate (Mohamed-Ahmed *et al.*, 2012 ▸; Maltezou, 2008 ▸). Drug resistance has been reported, leading to the use of more toxic and less effective compounds such as amphotericin B (Mohamed-Ahmed *et al.*, 2012 ▸; Maltezou, 2008 ▸). Alternative control strategies are needed because of the failure of current therapeutics and the presence of many reservoir hosts (http://www.who.int/tdr/diseases/leish/info/en/index.html).

*Leishmania* parasites are transmitted to humans by sandflies of the genus *Phlebotomus* and *Lutzomyia* in the Old and New Worlds, respectively (Collin *et al.*, 2009 ▸). To facilitate its blood meal, a sandfly injects saliva into the host, which enhances the survival of injected *Leishmania* promastigotes and the establishment of disease transmission (Titus & Ribeiro, 1988 ▸; Valenzuela *et al.*, 2001 ▸; Bezerra & Teixeira, 2001 ▸; Andrade *et al.*, 2007 ▸; Gomes *et al.*, 2008 ▸; Costa *et al.*, 2013 ▸). Additionally, the sandfly salivary proteins result in the production of antibodies that can serve as markers of exposure (Belkaid *et al.*, 1998 ▸). LJL143 from the saliva of the New World sandfly *L. longipalpis* is a known marker of exposure in zoonotic reservoirs (Teixeira *et al.*, 2010 ▸; Collin *et al.*, 2009 ▸). Structural studies of LJL143 were initiated as part of ongoing efforts to characterize the structure and functions of sandfly antigens. The production, crystallization and preliminary X-ray diffraction studies of LJL143 are presented.

## 2. Materials and methods   

### 2.1. Macromolecule production   

DNA coding for the mature LJL143 peptide and a C-terminal hexahistidine tag was codon-optimized based on the yeast codon preference (GenScript, Piscataway, New Jersey, USA). The synthesized gene included EcoRI and XbaI restriction sites for subcloning into the pPICZα A vector (Table 1 ▸). The plasmid DNA was linearized and transformed into *Pichia pastoris* strain X33 by electroporation as described previously (Zhan *et al.*, 2005 ▸). 20 colonies with the correct insert were screened for the expression of recombinant LJL143 protein with 0.5% methanol at 303 K for 72 h. The highest expressing colony was used to make a 1 l starter culture using buffered minimal glycerol medium (BMGY; 100 m*M* potassium phosphate pH 6.0, 1.34% yeast nitrogen base, 4 × 10^−5^% biotin, 1% yeast extract, 1% glycerol). Approximately 250 ml of the starter culture was added to a 14 l fermentation vessel containing 5 l basal salt medium (BSM; 26.7 ml 85% phosphoric acid, 0.93 g calcium sulfate, 18.2 g potassium sulfate, 14.9 g magnesium sulfate, 4.13 g potassium hydroxide and 40 g glycerol per litre). The BSM was maintained at pH 5 by the addition of 14% ammonium hydroxide. Antifoam 204 was added to minimize foaming. About 16 h into fermentation a dissolved-oxygen (DO) spike from the depletion of glycerol was observed, which prompted the initiation of a fed-batch phase. During the fed-batch phase, 50% glycerol was added to the culture at 15 ml per litre per hour for 6 h. In the last 2 h of the fed-batch phase, the pH of the culture was ramped from 1 to 6.0 and the temperature was reduced by 2 K. The fed-batch phase was followed by an 8 h methanol-adaptation phase during which the agitation was increased to 700 rev min^−1^ and a methanol feed was ramped to 11 ml per litre per hour, which was maintained until the end of fermentation.

**Figure 1:**
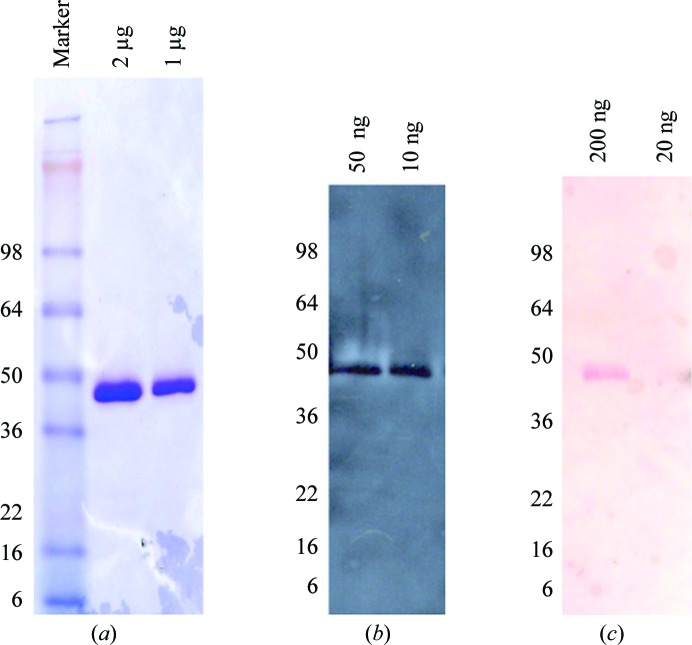
(*a*) The electrophoretic mobility of LJL143 reveals an ∼42 kDa protein. (*b*, *c*) Western blot of LJL143 with (*b*) anti-His-tag monoclonal antibody and (*c*) anti-LJL143 mouse serum.

After 72 h of induction, the culture medium containing the secreted LJL143 protein was separated from the yeast cells by centrifugation at 12 000*g* for 30 min and filtered with a 0.22 µm membrane filter. The clarified supernatant was buffer-exchanged into the binding buffer (500 m*M* sodium chloride, 5 m*M* imidazole, 20 m*M* Tris pH 8.0) using a 3 kDa hollow-fiber cartridge (GE Healthcare). LJL143 was purified by immobilized metal-ion affinity chromatography (IMAC) using a 5 ml HisTrap FF column (GE Healthcare). Nonspecifically bound proteins were removed by sequential steps of washing with 5, 10, 25 and 55 m*M* imidazole prior to protein elution with 500 m*M* imidazole. LJL143 was purified to ∼99% as assessed by Coomassie-stained SDS–PAGE (Fig. 1 ▸ *a*). The protein band was verified to be LJL143 by Western blotting using both anti-His-tag monoclonal antibody (Fig. 1 ▸ *b*) and mouse antiserum against LJL143 (Fig. 1 ▸ *c*).

### 2.2. Crystallization   

Recombinant LJL143 was buffer-exchanged and concentrated to 23 mg ml^−1^ in 50 m*M* sodium citrate buffer pH 5.0 using a 10K cutoff centrifugal concentrating device (Millipore). The protein concentration was measured by the absorbance at 280 nm prior to setting up crystallization experiments.

Initial crystallization screens were carried out using commercial sparse-matrix screens from Hampton Research (Crystal Screen, Crystal Screen 2 and Index) at 298 K by vapour diffusion in sitting drops (Table 2 ▸).

### 2.3. Data collection and processing   

The crystals were transferred for ∼30 s into cryoprotecting solution (0.1 *M* bis-tris pH 6.5, 28% PEG 2000 MME, 15% glycerol) and were flash-cooled directly in a stream of N_2_ gas at 113 K prior to collecting diffraction data. X-ray diffraction data were collected at the Baylor College of Medicine core facility using a Rigaku HTC detector (Table 3 ▸). The X-ray source was a Rigaku FR-E+ SuperBright microfocus rotating-anode generator with VariMax HF optics. A data set was collected from a single crystal using the *CrystalClear* (d*TREK) package (Pflugrath, 1999 ▸) and was processed using *MOSFLM* (Leslie, 2006 ▸).

## 3. Results and discussion   

**Figure 2:**
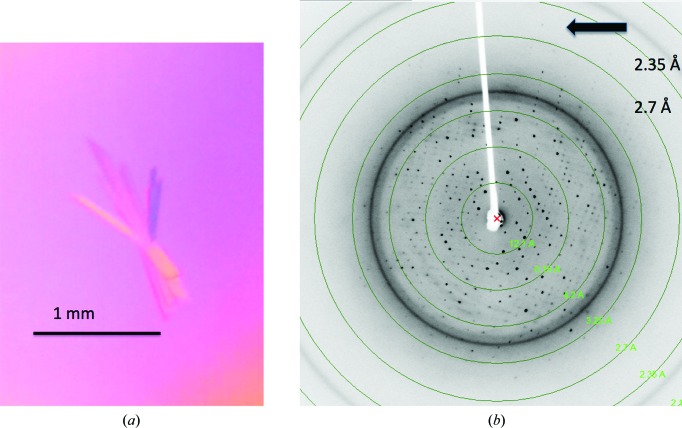
(*a*) Typical crystals of LJL143 are thin, flat rods of less than 0.01 mm on the smallest face. (*b*) A sample diffraction image of LJL143 crystals reveals visible spots to 2.6 Å resolution as indicated by the arrow.

The typical yield of LJL143 was 50 mg per litre of fermentation supernatant. The resulting protein had an electrophoretic mobility of 42 kDa. The predicted theoretical molecular mass of LJL143 is 33 594.92 Da. LJL143 has two predicted N-glycosylation sites at ^42^NQTH and ^241^NKTC, which may contribute to the almost 10 kDa increase in molecular mass. LJL143 crystals were grown using different polyethylene glycols (PEG) as precipitants. The best diffracting crystals were obtained after 48 h in the conditions detailed in Table 2 ▸. These crystals are illustrated in Fig. 2 ▸(*a*). Diffraction images reveal visible diffraction spots beyond 2.6 Å resolution (Fig. 2 ▸ *b*). The crystal belonged to the orthorhombic space group *P*2_1_2_1_2_1_, with approximate unit-cell parameters *a* = 57.5, *b* = 70.2, *c* = 79.5 Å (Table 3 ▸). Based on the estimated Matthews coefficient and solvent-content prediction (http://www.ruppweb.org/Mattprob), a monomer is expected in the asymmetric unit (Matthews, 1968 ▸; Kantardjieff & Rupp, 2003 ▸). This corresponds to a unit-cell volume of 321 104 Å^3^ (with an asymmetric unit of 80 276 Å^3^), a Matthews coefficient of 2.39 Å^3^ Da^−1^ and an estimated solvent content of 48.5%.

A *BLAST* analysis of LJL143 against the Protein Data Bank revealed no suitable homologues for molecular replacement. The closest structural homologue, 2-hydroxyhepta-2,4-diene-1,7-dioate isomerase from *Thermus thermophilus* (PDB entry 2dfu; RIKEN Structural Genomics/Proteomics Initiative, unpublished work), shares only 21% sequence identity over a 26% query coverage. There are only ten S atoms out of a total of 4668 atoms, and our attempts at phase determination by single-wavelength anomalous dispersion using S atoms at the Cu *K*α wavelength failed. Future studies will include other methods of experimental phase determination such as multi-wavelength anomalous dispersion phasing with selenomethionine-derivatized protein and heavy-atom derivatization for multiple isomorphous replacement (Ingram *et al.*, 1956 ▸; Perutz, 1953 ▸; Harker, 1956 ▸; Hendrickson & Ogata, 1997 ▸). LJL143 and other salivary-gland proteins play key roles in the transmission of leishmaniasis. The structure of LJL143 will give key insights into the underlying mechanisms of this poorly characterized family of proteins.

## Figures and Tables

**Table 1:** Macromolecule-production information

Source organism	*L. longipalpis*
DNA source	Synthetic
Forward primer	Not applicable: the gene was synthesized with an EcoRI restriction site and the sequence has a vector-derived N-terminal ‘EF’
Reverse primer	Not applicable: the gene was synthesized with an XbaI restriction site and the sequence includes a C-terminal His_6_ tag
Cloning vector	pPicZ A
Expression vector	pPicZ A
Expression host	*P. pastoris* X-33
Complete amino-acid sequence of the construct produced	EFDGDEYFIGKYKEKDETLFFASYGLKRDPCQIVLGYKCSNNQTHFVLNFKTNKKSCISAIKLTSYPKINQNSDLTKNLYCQTGGIGTDNCKLVFKKRKRQIAANIEIYGIPAKKCSFKDRYIGADPLHVDSYGLPYQFDQEHGWNVERYNIFKDTRFSTEVFYHKNGLFNTQITYLAEEDSFSEAREITAKDIKKKFSIILPNEEYKRISFLDVYWFQETMRKKPKYPYIHYNGECSNENKTCELVFDTDELMTYALVKVFTNPESDGSRLKEEDLGRGHHHHHH

**Table 2:** Crystallization

Method	Vapour diffusion in sitting drops
Plate type	Cryschem plate (Hampton Research)
Temperature (K)	298
Protein concentration (mgml^1^)	23
Buffer composition of protein solution	50m*M* sodium citrate buffer pH 5.0
Composition of reservoir solution	0.1*M* bis-tris pH 6.5, 28% PEG 2000 MME
Volume and ratio of drop	5.5l, 4.5:1
Volume of reservoir (l)	500

**Table 3:** Data collection and processing Values in parentheses are for the outer shell.

Diffraction source	Rigaku FR-E+ SuperBright
Wavelength ()	1.514
Temperature (K)	100
Detector	Rigaku R-AXIS HTC
Crystal-to-detector distance (mm)	150
Rotation range per image ()	0.5
Total rotation range ()	235
Exposure time per image (s)	45
Space group	*P*2_1_2_1_2_1_
*a*, *b*, *c* ()	57.39, 70.24, 79.58
, , ()	90, 90, 90
Mosaicity ()	1.04
Resolution range ()	44.42.6 (2.72.6)
Total No. of reflections	90330 (11114)
No. of unique reflections	10379 (1238)
Completeness (%)	99.8 (100)
Multiplicity	8.7 (9.0)
*I*/(*I*)	15.3 (2.4)
*R*_r.i.m._ (%)	3.1 (42.8)
Overall *B* factor from Wilson plot (^2^)	40.07
